# Cadaverine, a metabolite of the microbiome, reduces breast cancer aggressiveness through trace amino acid receptors

**DOI:** 10.1038/s41598-018-37664-7

**Published:** 2019-02-04

**Authors:** Tünde Kovács, Edit Mikó, András Vida, Éva Sebő, Judit Toth, Tamás Csonka, Anita Boratkó, Gyula Ujlaki, Gréta Lente, Patrik Kovács, Dezső Tóth, Péter Árkosy, Borbála Kiss, Gábor Méhes, James J. Goedert, Péter Bai

**Affiliations:** 1 Department of Medical Chemistry, Faculty of Medicine, University of Debrecen, Debrecen, 4032 Hungary; 2 Department of Dermatology, Faculty of Medicine, University of Debrecen, Debrecen, 4032 Hungary; 3 Department of Oncology, Faculty of Medicine, University of Debrecen, Debrecen, 4032 Hungary; 4 Department of Pathology, Faculty of Medicine, University of Debrecen, Debrecen, 4032 Hungary; 5 MTA-DE Lendület Laboratory of Cellular Metabolism, Debrecen, 4032 Hungary; 6 Kenézy Breast Center, Kenézy Gyula County Hospital, Debrecen, 4032 Hungary; 7 National Cancer Institute, National Institutes of Health, Bethesda, 20982 MD USA; 8 Research Center for Molecular Medicine, Faculty of Medicine, University of Debrecen, Debrecen, 4032 Hungary

## Abstract

Recent studies showed that changes to the gut microbiome alters the microbiome-derived metabolome, potentially promoting carcinogenesis in organs that are distal to the gut. In this study, we assessed the relationship between breast cancer and cadaverine biosynthesis. Cadaverine treatment of Balb/c female mice (500 nmol/kg p.o. q.d.) grafted with 4T1 breast cancer cells ameliorated the disease (lower mass and infiltration of the primary tumor, fewer metastases, and lower grade tumors). Cadaverine treatment of breast cancer cell lines corresponding to its serum reference range (100–800 nM) reverted endothelial-to-mesenchymal transition, inhibited cellular movement and invasion, moreover, rendered cells less stem cell-like through reducing mitochondrial oxidation. Trace amino acid receptors (TAARs), namely, TAAR1, TAAR8 and TAAR9 were instrumental in provoking the cadaverine-evoked effects. Early stage breast cancer patients, versus control women, had reduced abundance of the CadA and LdcC genes in fecal DNA, both responsible for bacterial cadaverine production. Moreover, we found low protein expression of *E. coli* LdcC in the feces of stage 1 breast cancer patients. In addition, higher expression of lysine decarboxylase resulted in a prolonged survival among early-stage breast cancer patients. Taken together, cadaverine production seems to be a regulator of early breast cancer.

## Introduction

Microbes that live on the surface or the cavities of the human body affect a large set of pathophysiological processes ranging from metabolic diseases to psychiatric disorders^1–4^ or neoplastic dieases^3,5–7^. The number of directly tumorigenic bacteria is extremely low (~10 species)^8^, however, dysbiosis is associated with cancers of the urinary tract^9^, cervix^10^, skin^11^, airways^12^, the colon^8^, lymphomas^13,14^, prostate^9^ and breast cancer^15–22^. Dysbiosis is often reflected as a loss of diversity of the microbiota (e.g.^16^). In colon carcinogenesis, immunogenic microbes probably promote the malignancy. However, the majority of the aforementioned cancers are located distantly from larger depots of microbes, hence, suggesting indirect induction or promotion mechanisms. Indeed, bacterial metabolites emerge as “endocrine” agents that are produced by the microbiome, are absorbed into the circulation, and exert their biological effects distantly.

Deconjugated estrogens^17,18^, secondary bile acids^23–28^, lipopolysaccharide^29^ or propionate (a short chain fatty acid (SCFA))^30^ were proposed to be involved in regulating transformation or cancer cell proliferation. Nonetheless, the molecular mechanisms, through which bacterial metabolites expert their effects are largely unknown. Deoxycholic acid (DCA) was shown to reprogram the hepatocyte secretome, thereby, promoting hepatocellular carcinoma^23,24^. Another secondary bile acid, lithocholic acid was shown to inhibit proliferation of breast cancer cells through inhibiting Warburg metabolism and endothelial-to-mesenchymal transition, as well as by enhancing antitumor immunity^26^. LCA exerted its antitumor effects through the TGR5 receptor^26^. Importantly, the latter study showed that in early stages of breast cancer bacterial LCA biosynthesis was decreased suggesting a loss of an antiproliferative bacterial metabolite^26^.

Cadaverine (CAD) is produced by the decarboxylation of lysine that is performed by lysine decarboxylase (LDC) enzymes. Human cells code and express LDC, but numerous bacterial species of the human microbiome also expresses LDC either in a constant (LdcC in the LDC operon) or in an inducible (CadA in the Cad operon) fashion^31,32^. Bacteria use diamines, like cadaverine or putrescine, generated by the decarboxylation of lysine or arginine, to buffer the pH of their environment^27^. The effects of cadaverine on cancer cells and its role in carcinogenesis is not characterized in detail. Therefore, we wanted to assess whether cadaverine can influence the behavior of breast cancer cells.

## Results

### Cadaverine treatment reduces metastasis formation in 4T1-grafted mice

**Figure 1:**
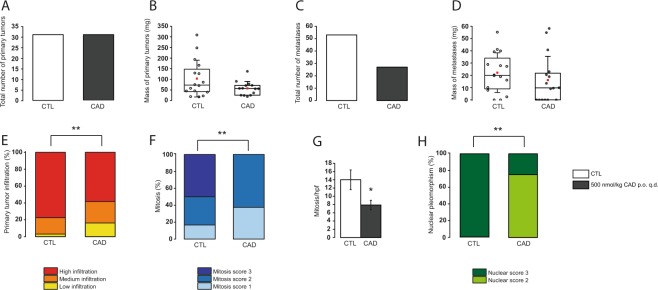
Cadaverine treatment reduces breast cancer aggressiveness *in vivo*. Female Balb/c mice were grafted with 4T1 cells as described and were treated with cadaverine (500 nmol/kg q.d. p.o.; CAD group) or vehicle (CTL) (n = 16/16) for 16 days before sacrifice. 6 samples from CTL group and 8 samples from cadaverine group underwent histological analysis. In CTL and CAD group (**A**) the number and (**B**) mass of primary tumors were counted and the (**C)** number and (**D)** mass of metastases were measured upon autopsy. (**E)** Upon autopsy, the infiltration rate of the primary tumor was scored. Significance (p = 0.002) was calculated using Fisher’s exact test with 2 × 3 Contingency Table. (**F–H**) Primary tumors were formalin-fixed and were embedded into paraffin, then sections were hematoxylin-eosin stained and were scored for (**F**) mitosis (p = 0.0031), (**G**) mitosis/hpf (p = 0.027) and (**H**) nuclear pleomorphism (p = 0.0097). Data is plotted as mean ± SEM. In box-whiskers charts (panels B and D) middle lines indicate the median, while red square symbol the mean. * and ** indicate statistically significant difference between CTL and CAD groups at p < 0.05 or p < 0.01, respectively. Significance was calculated using two-sample student t-test (two-tailed) except for panel E, F and H, where Chi-square or Fisher’s exact test was used.

As first step, we tested the effects of cadaverine supplementation (500 nmol/kg) to mice homotopically grafted with 4T1 breast cancer cells. Cadaverine supplementation did not alter the number of primary tumors that grew from the grafted cells (Fig. 1A), but there was a trend towards tumors with lower mass (Fig. 1B). In line with that, the number of metastases decreased (Fig. 1C) and, as with the primary tumors, there was a trend for smaller metastases in the cadaverine-treated mice (Fig. 1D). Importantly, cadaverine treatment decreased the invasivity of the primary tumors (Fig. 1E). Histological examination of the primary tumors revealed that cadaverine treatment decreased the rate of mitosis (Fig. 1F,G), the heterogeneity of nuclear morphology (Fig. 1H).

### Cadaverine administration does not impair breast cancer proliferation

**Figure 2:**
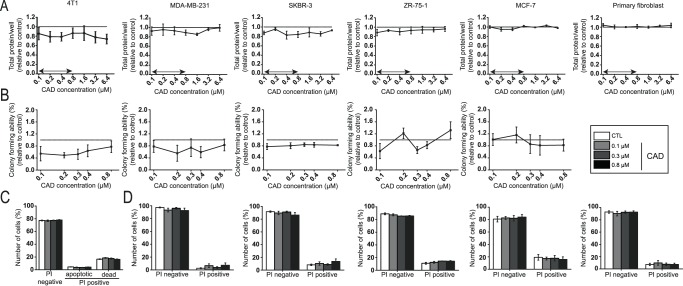
Cadaverine reduces the proliferation and colony forming ability of breast cancer cells. (**A**) 4T1 (n = 6 in octuplicates), MDA-MB-231 (n = 3 in octuplicates), SKBR-3 (n = 3 in octuplicates), ZR-75-1 (n = 3 in octuplicates) and MCF-7 (n = 3 in octuplicates) breast cancer cells and primary fibroblasts cells were treated with cadaverine in the concentrations indicated for 48 hours then total protein concentrations were determined in SRB assay. Values are expressed as fold change, where 1 means protein content in the control cells (indicated by a dotted line). (**B**) 4T1, MDA-MB-231, SKBR-3, ZR-75-1 and MCF-7 (n = 3 for each in one replicate) cells were treated with cadaverine in the concentrations indicated for 4 days. Colonies were then stained according to May-Grünwald-Giemsa and counted using Image J software (n = 3). (**C**) 4T1 cells (n = 3 in triplicates) were treated with cadaverine in the concentrations indicated for 48 hours. Cells were stained with Annexin-FITC-PI Apoptosis Kit and analyzed by flow cytometry (n = 4). (**D**) 4T1 (n = 3 in triplicates), MDA-MB-231 (n = 4 in triplicates), SKBR-3 (n = 4 in triplicates), ZR-75-1 (n = 3 in triplicates), MCF-7 (n = 3 in triplicates) and primary fibroblasts cells were treated with cadaverine in the concentrations indicated for 48 hours. Dead cells were stained with propidium iodide (PI) and analyzed by flow cytometry. Data is plotted as mean ± SEM. Significance was calculated using one-way ANOVA test. CAD – cadaverine, other abbreviations are in the text. Double-headed arrow mark the serum reference concentration of cadaverine on panel A.

We investigated whether cadaverine administration could influence the proliferation of cultured breast cancer cell lines. We used five different established breast cancer cell lines of which four were of human (MD-MBA-231, SKBR3, ZR-75-1 and MCF7), while one was of murine origin (4T1). The cadaverine concentration that we used corresponded to the reference concentration of cadaverine in human serum (100–800 nM)^33,34^. Cadaverine slowed proliferation of 4T1, MDA-MB-231 and SKBR-3 cells as measured in SRB assay (Fig. 2A) or in colony forming assays (Fig. 2B), although the changes were not statistically significant. Importantly, the same concentrations of cadaverine did not hinder the proliferation of non-transformed primary human skin fibroblasts (Fig. 2A). We assessed whether slower proliferation could be due to the toxicity of cadaverine to cells. The proportion of the PI positive cells did not increase upon cadaverine treatment (Fig. 2D), nor did the apoptotic fraction in 4T1 cells (Fig. 2C).

### Cadaverine induced a mesenchymal-to-epithelial (MET) transition and invasion

**Figure 3:**
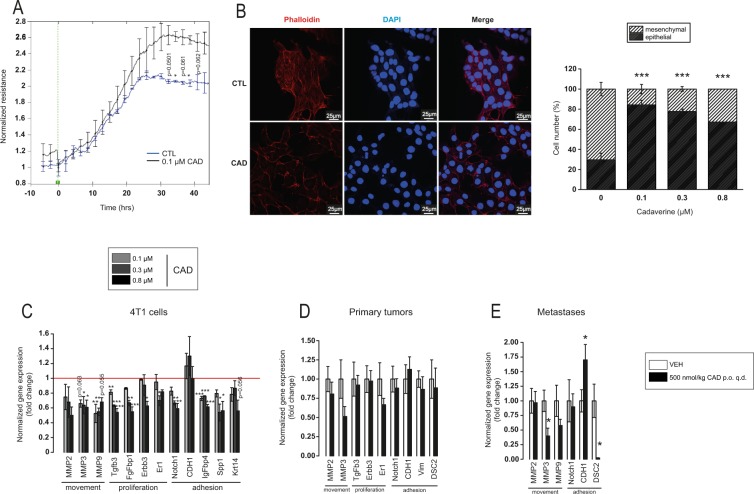
Cadaverine treatment reverses EMT of breast cancer cells. (**A**,**B**) In control and cadaverine-treated 4T1 cells (A) total cellular impedance was measured by ECIS (n = 2 in duplicate) (p = 0.0484 at 36 hrs. and 0.0487 at 40 hrs.) and (B) morphology of the actin cytoskeleton (representative picture of control and 0.1 µM cadaverine-treated cells) was assessed after Texas Red-X Phalloidin + To-Pro-3 staining (n = 2 in triplicates), ratio (%) of epithelial and mesenchymal cells was shown on bar chart. Significance was calculated using Chi-square test in Microsoft Excel (p = 2.97 × 10^−28^ between control and 0.1 µM cadaverine, p = 1.04 × 10^−18^ between control and 0.3 µM cadaverine, p = 3.97 × 10^−10^ between control and 0.8 µM cadaverine). (**C–E**) Expression of a set of genes involved in EMT were assessed by RT-qPCR in (**C**) 4T1 cells (n = 3), (**D**) primary tumors (n = 16/16) and in (**E**) metastases (n = 16/16). All data is expressed as fold change. The red line that equals to 1 (no change) indicate the average of the control samples. Data is plotted as mean ± SEM. On panel A and C-E significance was calculated using two-sample student t-test (two-tailed), while on panel B One-way Anova was used to calculate differences. Significant p values were shown in Supplementary Table 4. *^,^** and *** indicate statistically significant difference between control and treated groups at p < 0.05, p < 0.01 or p < 0.001 respectively. CAD – cadaverine, other abbreviations are in the text.

We assessed whether cadaverine treatment can revert mesenchymal-like cancer cells to epithelial-like cells. First, we performed a measurement in the ECIS system in which 0.1 µM cadaverine increased resistance, suggesting better adherence of cells (Fig. 3A). To verify these findings, we stained cells with Phalloidin-Texas Red to visualize the arrangement of the actin cytoskeleton. Cadaverine treatment changed the fibroblast-like morphology of the 4T1 cells to a rather cobblestone-like morphology (Fig. 3B) that is characteristic for epithelial cells^26^. Treatment of MDA-MB-231 and SKBR-3 breast cancer cell lines with cadaverine led to similar morphological changes (Fig. S1A,D).

To gain insight into the molecular mechanism through which MET takes place we performed an RT-qPCR screen on EMT genes. The assay revealed differential expression of 11 genes after cadaverine treatment (Table 1), most were suppressed. MMP2, MMP3 and MMP9 support movement; Tgfb3, FgFbp1, Erbb3 and Er1 support proliferation; while Krt14, Notch1, CDH1, IgFbp4 and Spp1 support cell adhesion (Fig. 3C–E).

**Table 1:** List of EMT genes differentially regulated upon cadaverine treatment.

Abbreviation	Gene name	Category
MMP2	Matrix Metalloproteinase 2	Extracellular matrix and cell adhesion
MMP3	Matrix Metalloproteinase 3	
MMP9	Matrix Metalloproteinase 9	
Krt14	Keratin 14	
CDH1	E-cadherin	
Spp1	Secreted Phosphoprotein 1	
FgfBp1	Fibroblast Growth Factor Binding Protein 1	Cell growth and proliferation
Notch1	Notch 1	
Tgfb3	Transforming Growth Factor Beta 3	
Erbb3	Human Epidermal Growth Factor Receptor 3	
Esr1	Estrogen Receptor 1	
IgfBp4	Insulin Like Growth Factor Binding Protein 4	

**Figure 4:**
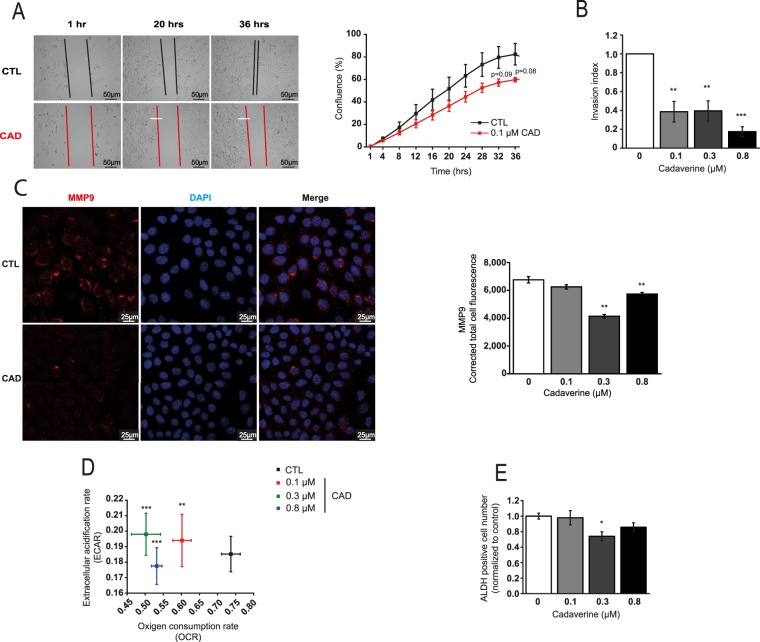
Cadaverine treatment attenuate movement, invasion ability, mitochondrial oxidation and stem-ness of 4T1 cells. (**A**) 4T1 cells were treated with cadaverine in the concentrations indicated for 48 hours after scratching a 4T1 cell layer. Subsequent closure of the wound was assessed by the JULI-Br live cell analyzer system (n = 2). Significance was calculated using two-sample student t-test (two-tailed). (**B**) 4T1 cells were treated with cadaverine for 48 hours and subsequently invasion capacity of the cells was measured using the Corning Matrigel invasion chamber. Cells were counted using the Opera HCS system and invasion index was calculated (p = 0.003 both between control and 0.1 µM cadaverine, and control and 0.3 µM cadaverine, p = 4.14 × 10^−4^ between control and 0.8 µM cadaverine). (**C**) 4T1 cells were treated with vehicle and cadaverine for 48 hours, then cells were stained for MMP9 and nucleus (DAPI) and sections were analyzed by confocal microscopy using a Leica SP8 confocal system. MMP9 content was calculated from the total cellular fluorescence measured by the Image J software (p = 0.001 between both control and 0.3 µM cadaverine and control and 0.8 µM cadaverine). (**D**) 4T1 cells were treated with vehicle and cadaverine for 48 hours (n = 3 in 24 replicates), then cells were subjected to a Seahorse XF96 analysis. Oxygen consumption rate (OCR) and extracellular acidification rate (ECAR) were measured and plotted (p_OCR_ = 0.006 between control and 0.1 µM cadaverine, p_OCR_ = 1.14 × 10^−6^ between control and 0.3 µM cadaverine, p_OCR_ = 1.8 × 10^−5^ between control and 0.8 µM cadaverine). (**E**) 4T1 cells were treated with vehicle and cadaverine for 48 hours (n = 3 in triplicates), then cells were subjected to an Aldefluor assay (p = 0.035 between control and 0.3 µM cadaverine). Data is plotted as mean ± SEM. Significance was calculated using One-way Anova. *^,^** and *** indicate statistically significant difference between control and treated groups at p < 0.05, p < 0.01 or p < 0.001 respectively. CAD – cadaverine, other abbreviations are in the text.

In line with these observations, cadaverine-treated cells were slower in migrating to open areas in scratch assays (Fig. 4A) and also performed worse in Boyden-chamber transmigration tests (Fig. 4B). These data were further supported by the observation that MMP9 expression was suppressed by cadaverine treatment in 4T1 cells (Fig. 4C), as well as in MDA-MB-231 and SKBR-3 breast cancer cell lines (Fig. S1B,E). We assessed metabolic changes evoked by cadaverine administration using the Seahorse flux analyzer. Cadaverine treatment reduced glycolytic flux (Fig. 4D) that is a characteristic of breast cancer stromal cells^35^. Therefore, we assessed the “stem-ness” of 4T1 cells using the aldefluor assay and found a mild reduction in cancer cell stem-ness (Fig. 4E). We found similar reductions in cancer cell stem-ness in MDA-MB-231 cells upon cadaverine treatment (Fig. S1C). We did not detect changes in lipid oxidation (Fig. S2).

### Cadaverine exert its beneficial effects through Trace Amino Acid Receptors (TAARs)

**Figure 5:**
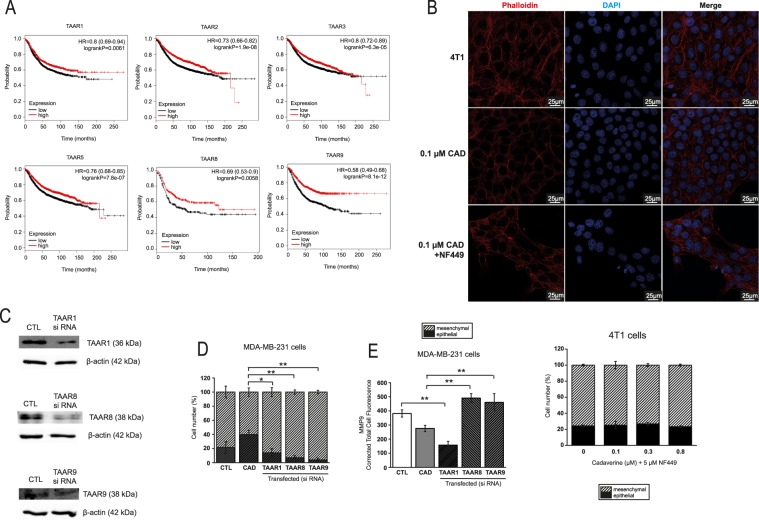
TAARs are important in mediating the effects of cadaverine. (**A**) Patient data was accessed at kmplot.com. Kaplan-Meier plots show the correlation between the mRNA expression of human TAARs and survival in breast cancer. (**B**) 4T1 cells were treated with, 100 nM cadaverine (48 hours) or 100 nM cadaverine (48 hours) in combination with 5 µM NF449 G protein inhibitor (last 48 hours of cadaverine treatment), cells were stained with Texas Red-X Phalloidin- and DAPI then cells were assessed by confocal microscopy (n = 2 in triplicates). (**C**) MDA-MB-231 cells were transfected with control si RNA or TAAR si RNA. The silencing efficacy was measured by Western blot (n = 2). (**D**,**E**) MDA-MB-231 cells were treated with vehicle or 0.3 μM cadaverine and transfected with control si RNA or TAAR si RNA. After 48 hours of incubation (**D**) TexasRed-X Phalloidin immunocytochemistry (p = 0.04 between cadaverine and TAAR1, p = 0.007 between cadaverine and TAAR8, p = 0.003 between cadaverine and TAAR9) (n = 1 in triplicates), and (**E**) MMP9 immunocytochemistry (p = 0.006 between control and TAAR1; p = 0.001 between cadaverine and TAAR8; p = 0.004 between cadaverine and TAAR9) (n = 1 in triplicates) were performed. CAD – cadaverine, other abbreviations are in the text.

The trace amino acid receptor family serve as receptors for cadaverine^36^. Although, most studies on TAAR focused on olfaction^36^, a study linked TAAR1 to breast cancer^37^. Indeed, higher expression of TAAR1, TAAR2, TAAR4, TAAR5, TAAR8 (in ER- cases) and TAAR9 provided better survival in breast cancer (Fig. 5A, Table 2). First, as TAAR receptors are G protein-dependent receptors^36^ we assessed their involvement by treating 4T1 cells with NF449, a Gsα-subunit-selective G-protein antagonist, a treatment that abolished the anti-EMT effect of cadaverine (Fig. 5B). Next, we silenced TAAR1, TAAR8 and TAAR9 in MDA-MB-231 cells (Fig. 5C). The silencing of TAAR1, TAAR8 and TAAR9 prevented the cadaverine-elicited mesenchymal-to-epithelial transition and the silencing of TAAR8 and TAAR9 prevented the cadaverine-induced decrease in MMP9 expression (Fig. 5D,E).

**Table 2:** Connection between TAARs and Breast Cancer Patient survival.

TAAR1 (1553930_at)	HR (Hazard Ratio)	P-value (Log Rank Test)
*All Breast Cancers, N* = *1764*	0.8	**6.1 × 10** ^− **3**^
*ER*(−), *N* = *516*	0.77	0.054
*ER*(+), *N* = *1248*	0.83	0.052
*Basal subtype, N* = *360*	0.66	**0.011**
*Luminal B, N* = *407*	0.67	**0.01**
**TAAR2** (**221394_at)**		
*All Breast Cancers, N* = *3951*	0.73	**1.90 × 10** ^− **8**^
*ER*(−), *N* = *869*	0.65	**6.20 × 10** ^− **5**^
*ER*(+), *N* = *2061*	1.07	**0.4**
*Basal subtype, N* = *618*	0.66	**1 × 10** ^− **3**^
*Luminal B, N* = *1149*	0.66	**3.10 × 10** ^− **5**^
**TAAR3** (**221393_at)**		
*All Breast Cancers, N* = *3951*	0.8	**6.30 × 10** ^− **5**^
*ER*(−), *N* = *869*	0.69	**4.5 × 10** ^− **4**^
*ER*(+), *N* = *3082*	0.86	**0.018**
*Basal subtype, N* = *618*	0.76	0.036
*Luminal B, N* = *1149*	0.67	**4.20 × 10** ^− **5**^
**TAAR5** (**221459_at)**		
*All Breast Cancers, N* = *3951*	0.76	**7.80 × 10** ^− **7**^
*ER*(−), *N* = *869*	0.75	**8.3 × 10** ^− **3**^
*ER*(+), *N* = *3082*	0.8	**6.00 × 10** ^− **4**^
*Basal subtype, N* = *618*	0.73	**0.016**
*Luminal B, N* = *1149*	0.62	**1.00 × 10** ^−− **6**^
**TAAR8 (1553552_at)**		
*All Breast Cancers, N* = *1764*	0.94	0.42
*ER*(−), *N* = *516*	0.69	**5.8 × 10** ^− **3**^
*ER*(+), *N* = *1248*	1.1	0.31
*Basal subtype, N* = *360*	0.64	**7.8 × 10** ^− **3**^
*Luminal B, N* = *407*	0.97	0.85
**TAAR9 (1553066_at)**		
*All Breast Cancers, N* = *1764*	0.58	**8.10 × 10** ^− **12**^
*ER*(−), *N* = *516*	0.55	**1.40 × 10** ^− **5**^
*ER*(+), *N* = *1248*	0.6	**2.30 × 10** ^− **7**^
*Basal subtype, N* = *360*	0.5	**4.80 × 10** ^− **5**^
*Luminal B, N* = *407*	0.69	**0.017**

We assessed the available databases to collect data on TAAR1, TAAR8 and TAAR9. TAAR1 and TAAR9 expression did not show any major association with carcinogenesis or breast cancer subtypes in contrast to TAAR8. TAAR8 expression decreased in pre-cancerous lesions, such as hyperplastic enlarged lobular units (HELUs) as compared to normal terminal ductal lobular units (TDLUs)^38^. In line with that, TAAR8 expression decreased in breast cancer^39^. Decreases in TAAR8 expression was different between the histological subtypes of breast cancer. TAAR8 expression was lower in triple negative breast cancers as compared to non-triple negative breast cancers. There was a trend for lower TAAR8 expression in DCIS when compared to healthy tissue^40^, TAAR8 expression was lower in ductal invasive breast cancer as compared to healthy ducts^41^ and there was a trend for lower TAAR8 expression in lobular invasive breast cancer as compared to healthy lobes^41^. Taken together, apparently, in early stage of breast cancer TAAR8 expression decreases that is more pronounced in triple negative breast cancers.

### Cadaverine biosynthesis is suppressed in breast cancer

**Figure 6:**
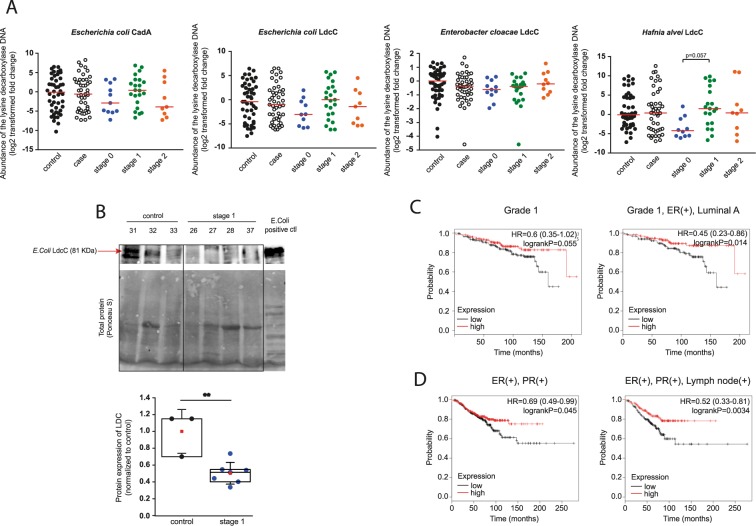
Cadaverine biosynthesis is suppressed in early stages of breast cancer. (**A**) Human fecal DNA samples were collected from 48 patients with different stages of breast cancer, and from 48 healthy patients. The abundance of DNA coding for CadA and LdcC of the indicated bacterial species were determined in the fecal DNA samples by RT-qPCR. Median values are indicated by a line. (**B**) Human fecal samples were collected from stage 1 breast cancer patients (n = 7) and from healthy (control) subjects (n = 3). The *E. coli* LdcC protein level was determined using Western blot. Hereby we show a representative image. Band intensity was normalized to total protein content assessed by ponceau-S staining of whole blots. Box chart of LDC protein expression from fecal samples (p = 0.003). Data was normalized to the mean of control samples. Blots were routinely cut as the representative blot. (**C**,**D**) Patient data was accessed at kmplot.com. Kaplan-Meier plots the correlation between the mRNA expression of human LDC and survival in breast cancer. Graphs show the correlation between LDC and disease survival in different forms and stages of the disease. Those arrays were also included where ER status was deducted from gene expression data. **Indicate statistically significant difference between control and treated groups at p < 0.01. Significance was calculated using two-sample student t-test (two-tailed). All abbreviations are in the text. Values on (**A**) were log2 transformed.

To get an insight whether intestinal cadaverine biosynthesis is modified in breast cancer patients, we assessed the abundance of the DNA coding for LdcC and CadA in human fecal DNA from the experimental cohort described in^16^. We designed primers for known CadA and LdcC genes in different bacteria. When comparing healthy individuals and breast cancer patients we observed slightly decreased abundance of *Escherichia coli* CadA and also *E. coli*, *Enterobacter cloacae* and *Hafnia alvei* LdcC DNA in breast cancer patients (Fig. 6A). Decreased CadA and LdcC abundance was more pronounced in clinical stage 0 patients as compared to the pool of all patients (Fig. 6A). Subsequently, we assessed the protein levels of *E. coli* LdcC protein in feces by Western blotting. In the feces of stage 1 patients LdcC protein levels were markedly lower than the levels in the feces of healthy subjects (Fig. 6B), in line with the lower fecal DNA abundances.

We assessed the GEO database to study LDC expression in human breast cancer. There was no difference in LDC mRNA expression between control and breast cancer cases^42–44^ or in LDC expression of the normal breast epithelium and cancer epithelium in patients^45^. Rather as an exception, LDC expression was lower in basal-like breast cancer as compared to control (normal) breast epithelium of non-diseased individuals^46^.

Finally, we assessed how expression of LDC in humans affects the outcome of breast cancer using the kmplot.com database (the acquired data is represented in Table 3). Although, differences in LDC expression did affect overall survival of the patients, in grade 1 patients higher expression of LDC was associated with significantly longer survival than lower expression of LDC (Fig. 6C). Interestingly, while LDC expression did not affect survival in ER- PR- patients, higher LDC expression correlated with better survival in ER+ PR+ patients (Fig. 6D).

**Table 3:** Breast cancer survival and Lysine decarboxylase.

Lysine decarboxylase (201744_s_at)	HR (Hazard Ratio)	P-value (Log Rank Test)
All Breast Cancers, N = 3951	1.07	0.23
ER(+), PR(+), N = 577	0.69	**0.045**
ER(+), PR(+), Lymph node(+), N = 344	0.52	**3.4 × 10** ^− **3**^
ER(+), PR(+), Lymph node(−), N = 228	0.76	0.39
ER(−), PR(−), N = 298	0.94	0.74
ER(−), PR(−), Lymph node(+), N = 127	0.76	0.32
ER(−), PR(−), Lymph node(−), N = 167	0.89	0.69
ER(−), PR(−), HER2(−), N = 198	1.01	0.98
ER(+), Luminal A, N = 1933	1.15	0.1
ER(+), Luminal A, Grade 1, N = 267	0.45	**0.014**
ER(+), Luminal B, N = 1149	1.11	0.27
ER(+), Luminal B, Grade 1, N = 56	1.73	0.39
Grade 1, N = 345	0.6	**0.055**
Grade 2, N = 901	0.83	0.13
Grade 3, N = 903	1.06	0.59
Basal subtype, N = 618	1.18	0.19
Luminal A, N = 1933	1.15	0.1
Luminal B, N = 1149	1.11	0.27
ER(+), HER2(+), N = 156	0.96	0.9
ER(−), HER2(+), N = 96	1.57	0.16

## Discussion

In this study we assessed how cadaverine affects the behavior of breast cancer cells. Cadaverine is an ill-characterized biogenic amine as compared to – for example – putrescine that is characterized as a pro-carcinogenic agent^31,47^. Increased levels of diamines are in positive correlation with carcinogenesis; serum level of diamines are higher in cancer patients than in healthy individuals^33,34,48^. Moreover, surgical removal of tumors normalize serum diamine levels^33,34^. Based on these observations, polyamines were suggested as cancer markers^49^, although their efficiency was not proven^47^. While in the available literature levels of putrescine were equivocally higher in various cancer patients than in healthy individuals^33,34,48^, cadaverine levels were more variable among the studies, some reporting increases in cadaverine levels^33,34,48^, others with trends for decreases^50^. Breast cancer patients have not been evaluated.

**Figure 7:**
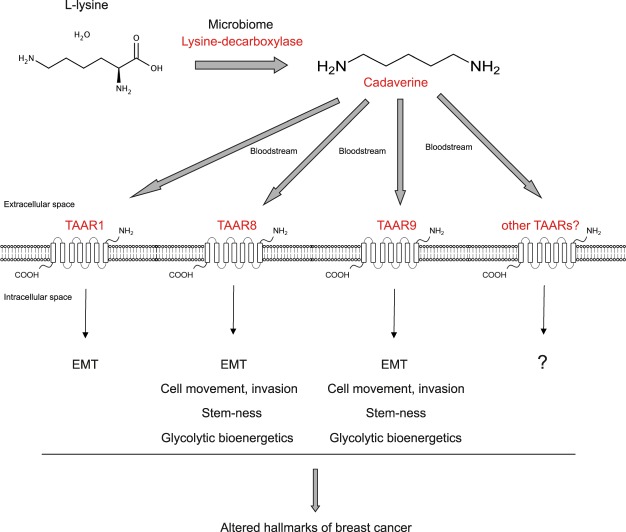
Scheme of the mechanism of cadaverine treatment.

Our data suggests that cadaverine has a tumor suppressor role in breast cancer in concentrations corresponding to the human reference range^33,34^. Cadaverine exerted its effects through inhibiting EMT, cellular movement, chemotaxis and metastasis (Fig. 7). These concentrations are much lower than the ones used in previous *in vitro* studies (10 µM in^51^ or 100 µM in^52^). Moreover, similarly to another cytostatic bacterial metabolite, lithocholic acid^26^, cadaverine, in the concentrations corresponding to its reference concentration, did not exert its cytostatic effects on primary fibroblasts suggesting that its effects are specific for tumor cells.

Cadaverine was shown to activate TAARs, moreover, a study linked TAAR1 to breast cancer^37^. Our results pointed out the possible involvement of TAAR1, TAAR2, TAAR3, TAAR5, TAAR8 and TAAR9 in bringing about the anticancer effects of cadaverine. High expression of these receptors in tumors associated with better survival. The lack of commercially available reagents limited us in validating these findings, we were able to test TAAR1, TAAR8 and TAAR9. All receptors were involved in regulating the mesenchymal-to-epithelial transition, however, only TAAR8 and TAAR9 modulated MMP9 expression (Fig. 7). Furthermore, the expression of TAAR8 was reduced in precancerous states and was gradually downregulated as the severity of the disease worsened; we have not found such correlations with TAAR1 or TAAR9. Available metadata suggest that TAAR8 has strong inverse correlation with breast cancer severity. Due to the limited availability of reagents, we cannot exclude that other TAARs could be equally involved in regulating cadaverine-evoked effects besides the ones we identified.

Our data suggest that bacterial cadaverine biosynthesis decreases in the gut in early stage breast cancer, resulting in lower production of an anti-cancer bacterial metabolite. In that sense, lower cadaverine production is in line with the lower production of another cytostatic microbial metabolite, lithocholic acid^26^. On a broader scale, these observations suggest that the simplification of the gut microbiome in early stage breast cancer^16,26^ leads to a decreased production of antiproliferative bacterial metabolites like cadaverine or lithocholic acid^26^. This observation is further supported by the observation that there is a positive correlation between breast cancer incidences or recurrence and cumulative antibiotic use^53–58^. Antibiotic exposure increased breast cancer risk in a dose-dependent fashion, nevertheless, these are correlative studies, and the chance of uncontrolled confounding is high and certain studies have found no association^53,54,56–58^. Apparently, the cumulative antibiotic dose is in correlation with increases in breast cancer frequency regardless of antibiotic class, although, the strongest effects were associated with the intermitting use of tetracyclins and macrolids^55^.

The most drastic changes to the microbiome was associated with *in situ* carcinoma or stage I carcinoma, in later stages cadaverine production re-increased and reached levels similar to the level of the controls. These findings are similar to our previous observations, in breast cancer the abundance of the cadaverine-producing bacteria was the lowest in *in situ* carcinoma and stage I carcinoma patients^26^. Nevertheless, the *in silico* data that we gathered for TAAR8 suggest that in parallel to the simplification or dysbiosys of the microbiome in tumors the expression of the cadaverine-sensing apparatus is probably downregulated.

There are other sources of cadaverine. Milk ducts in the breast are colonized by bacteria and the duct microbiome changes upon breast cancer^19–22^ that may contribute to changes in the breast pool of cadaverine. In addition to these, human cells also synthesize cadaverine. High expression of human LDC prolongs survival in early stage breast cancer patients, further supporting the potential anti-cancer properties of cadaverine. Nevertheless, the share of these sources (healthy breast tissues vs. tumor vs. host vs. gut) in cadaverine production is not explored.

This study puts cadaverine on the list of bacterial metabolites that govern carcinogenesis. Lithocholic acid^26^ and cadaverine can suppress cancer hallmarks of breast cancer cells. In hepatocellular carcinoma, lipopolysaccharide^29^ and deoxycholic acid (DCA)^23^ have been identified as promoters, while propionate is an inhibitor^30^. These observations support the hypothesis that bacterial metabolites influence carcinogenesis in organs remote from the gut. Nevertheless, this field requires considerably more research to understand the interactions between the microbiome and distantly located tumors.

## Methods

All methods were performed according to the relevant guidelines.

### Chemicals

Chemicals, among them, cadaverine were from Sigma-Aldrich (St. Louis, MI, USA) unless otherwise stated.

### Cell culture

4T1 murine breast cancer cells, ZR-75-1 human breast cancer cells were maintained in RPMI-1640 (Sigma-Aldrich, R5886) medium containing 10% FBS, 1% penicillin/streptomycin, 2 mM L-glutamine and 1% pyruvate at 37 °C with 5% CO_2_.

MDA-MB-231 and SK-BR-3 human breast cancer cells were maintained in DMEM (Sigma-Aldrich, 1000 mg/l glucose, D5546) containing 10% FBS, 1% penicillin/streptomycin, 2 mM L-glutamine and 10 mM HEPES at 37 °C with 5% CO_2_.

MCF7 human breast cancer cells were maintained in MEM (Sigma-Aldrich, M8042) medium containing 10% FBS, 1% penicillin/streptomycin, 2 mM L-glutamine and 10 mM HEPES at 37 °C with 5% CO_2_.

Human primary fibroblast cells were maintained in DMEM (Sigma-Aldrich, 1000 mg/l glucose, D5546) containing 20% FBS, 1% penicillin/streptomycin, 2 mM L-glutamine and 10 mM HEPES at 37 °C with 5% CO_2_.

### Transfections

Transfections were performed as in^59^.

### Cell proliferation assays

Sulphorhodamine B assay and colony forming assays were performed as in^26,60^.

### Detection of cell death

For the detection of cell death we used simple propidium iodide (PI, Biotium, Fremont, CA, 40016) uptake assays (as in^61^), while to differentiate between apoptosis and necrosis we used an Annexin V + PI double staining assay kit (Invitrogen, Oregon, USA, V13242).

### Electric Cell-substrate Impedance Sensing (ECIS)

ECIS assays were performed similarly to^26^. ECIS measurements are used to assess cell-to-cell and cell-to-surface connections.

### Immunocytochemistry

Immunocytochemistry was performed similarly to^26,62^.

### Bacterial LdcC and CadA quantitation

The human fecal DNA library from breast cancer patients was published in^16^. For the determination of the abundance of Lysine decarboxylase coding DNA in human fecal DNA samples 10 ng of DNA was used for qPCR reactions. Primers are listed in Supplementary Table 1. The amplicons were subsequently sequenced using the same primers as listed in Supplementary Table 1.

### mRNA isolation and quantitation

Reverse transcription-coupled PCR (RT-qPCR) was performed similarly to^63^.

### Fecal protein sample preparation

RIPA buffer (50 mM Tris, 150 mM NaCl, 0.1% SDS, 1% Triton X-100, 0.5% sodium deoxycholate, 1 mM EDTA, 1 mM Na_3_VO_4_, 1 mM NaF, 1 mM PMSF, protease inhibitor cocktail) was used to lyse cells of fecal samples. Samples were sonicated (Qsonica Q125 Sonicator, Newtown, Connecticut) 3 times for 30 seconds with 50% amplitude. After centrifugation, 25 µl 5 X SDS sample buffer (50% glycerol, 10% SDS, 310 mM Tris HCl, pH 6.8, 100 mM DTT, and 0.01% bromophenol blue) and 8 µl β-mercaptoethanol were added to every 100 µl supernatant. Samples were heated 10 minutes at 96 °C and kept on ice until loading.

### SDS-PAGE and Western blotting

SDS PAGE and Western blotting was performed as in^63^.

### Scratch assay

Scratch assays were performed as in^26^. Scratch assays were employed to assess cell movement.

### Invasion

Matrigel invasion assay was carried out on 4T1 cells using Corning BioCoat Matrigel Invasion Chamber (354480). 4T1 cells were seeded in the chambers (50000 cells/well) in serum free medium, and were grown overnight. Cells were then treated with different concentration of cadaverine (0.1 µM, 0.3 µM, 0.8 µM). The lower chamber contained full 4T1 medium with 100 ng/ml SDF1-alpha (Sigma, SRP4388) as chemoattractant. After 48 hours of cadaverine treatment cells were prepared according to the manufacturer’s instructions and stained with Hematoxylin-Eosin (VWR, 340374T and 341972Q) dye. Cells were then pictured with Opera Phoenix High Content Screening System and pictures were analyzed using Harmony 4.6 Software. Invasion index was calculated from the percentage of invading cells through Matrigel membrane and control membrane.

$$ \% \,Invasion=\frac{Mean\,of\,cells\,invading\,through\,Matrigel\,insert\,membrane}{Mean\,of\,cells\,invading\,through\,Control\,insert\,membrane}\ast 100$$

$$Invasion\,index=\frac{ \% \,Invasion\,Test\,Cell}{ \% \,Invasion\,Control\,Cell}$$

### Seahorse metabolic flux analysis

Changes to oxygen consumption rate (OCR, a readout of mitochondrial oxidation) and in pH (ECAR, a readout of lactate production and glycolytic flux) after cadaverine treatment were performed similarly to^26^.

### Determination of lipid peroxidation (TBARS)

TBARS assay was performed to assess lipid peroxidation as in^64^.

### Aldefluor assay

Aldefluor assay for assessing stem-ness was performed similarly to^65^.

### Animal study

Animal studies were conducted similarly to^26^.

Animal experiments were approved by the Institutional Animal Care and Use Committee at the University of Debrecen and the National Board for Animal Experimentation (1/2015/DEMÁB) and were carried out according to the NIH guidelines (Guide for the care and use of laboratory animals) and applicable national laws. Animal studies are reported in compliance with the ARRIVE guidelines^66,67^.

We used BALB/c female mice (4 months of age, 20–25 g). Animals were bred in the “specific pathogen free” zone of the Animal Facility at the University of Debrecen, and kept in the “minimal disease” zone during the experiments. 4 mice were housed in one cage (standard block shape 365 × 207 × 140 mm, surface 530 cm^2^; 1284 L Eurostandard Type II. L from Techniplast). Dark/light cycle was 12 h, and temperature was 22 ± 1 °C. Mice had ad libitum access to food and water (sterilized tap water). A total of 32 female mice were used in the study, 16 randomly selected control and 16 cadaverine fed mice. The study was performed in two runs at two different occasions, each run comprising of 8 vehicle-treated and 8 cadaverine-treated mice.

Tumor was formed in mice by the grafting of 4T1 cells. 4T1 cells were suspended (2 × 10^6^/mL) in ice cold PBS-Matrigel (1:1, Sigma-Aldrich). 16 female BALB/c mice received 50 µL injections to the inguinal fat pads below the lower abdominal nipples on both sides (10^5^ cells/injection site).

Animals received daily oral cadaverine treatment. Cadaverine stock was prepared in sterilized tap water at 100x concentration (15 mM) and the stock was stored at −20 °C. Cadaverine stock was diluted each day to a working concentration of 150 µM in sterile tap water before the treatment. Animals received a daily oral dose of 100 µl/30 g bodyweight from cadaverine solution (8 mice) or vehicle (sterilized tap water, 8 mice). Researchers administering cadaverine and vehicle solutions were blinded. Treatment was carried out every day during the morning hours between 9 am and 11 am. Mice were sacrificed on day 14 post grafting.

During autopsy primary tumors were scored based on their infiltration rate into surrounding tissues based on macroscopic appearance of the tumor. “Low infiltration” class means that primer tumor remained in the mammary fat pads without any attachment to muscle tissues. In case the tumor mass attached to the muscle tissue but did not penetrate to the abdominal wall, it classified as a “medium infiltration” tumor. If the tumor grew into the muscle tissue and totally penetrated the abdominal wall, it was scored as a “high infiltration” tumor.

Both primary and metastatic tumor masses were removed from mice and were measured on analytical balance in preweighed Eppendorf tubes.

### Human studies

We assessed the abundance of the bacterial DNA coding for lysine decarboxylase in human fecal DNA samples. The human fecal samples were collected from healthy women and breast cancer patients by collaborators at the National Cancer Institute (NCI), Kaiser Permanente Colorado (KPCO), and the Institute for Genome Sciences at the University of Maryland School of Medicine, and RTI International. The study protocol and all study materials were approved by the Institutional Review Boards at KPCO, NCI, and RTI International (IRB number 11CN235). The primary study results were published in^18^. All methods were performed in accordance with the relevant guidelines and regulations.

We obtained informed consent from study participants.

Another cohort was used to assess LdcC protein in the feces of healthy volunteers and breast cancer patients. The collection and biobanking of feces was authorized by the Hungarian national authority (ETT). Patients and healthy volunteers meeting the following criteria were excluded from the study according to the corresponding national guideline for fecal transplantation^68^: (1) has previous history of breast cancer or had been operated due to neoplasia, (2) has a disease of unknown origin, (3) has chronic contagious disease, (4) had contagious diarrhea 6 months prior to enrollment, (5) taken antibiotics in the 6 months prior to enrollment, (6) had chemotherapy, biological therapy or immunosuppressive therapy 6 months prior to enrollment, (7) used intravenous drugs 12 months prior to enrollment, (8) had piercing, tattooing, acupuncture or other endangering behavior or action 12 months prior to enrollment, (9) exposition to an allergen to which the enrolled individual had been sensitized to, (10) underwent colonoscopy 12 months prior to enrollment. First morning feces was sampled; samples were frozen and deposited in the biobank two hours after defecation. Samples were stored at −70 °C until subsequent use.

### Database screening

The kmplot.com database^69^ was used to study the link between gene expression levels and breast cancer survival in humans. The association of known mutations with breast cancer was retrieved from www.intogen.org/. The sequence of the *CadA and LdcC* ORFs were retrieved from the KEGG (www.genome.jp/kegg/)database. We assessed the NCBI GEO Profiles with the term “LDC and breast cancer” and the GENT database with the keywords “TAAR1”, “TAAR8” and “TAAR9”.

### Statistical analysis

We used two tailed Student’s *t*-test for the comparison of two groups unless stated otherwise. Fold data for human fecal DNA assessment were log_2_ transformed to achieve normal distribution. For multiple comparisons one-way analysis of variance test (ANOVA) was used followed by Tukey’s honestly significance (HSD) post-hoc test. Data is presented as average ± SEM unless stated otherwise. Texas Red-X Phalloidin-labelled fluorescent pictures were analyzed using Image J or Cell Profiler 2.0 followed by Advanced Cell Classifier 3.0. FACS results were analyzed using Flowing Software 2.0. Statistical analysis was done using Origin 8.6 software unless stated otherwise.

## Supplementary information

Supplelemtary information 1

Supplelemtary information 2

Supplelemtary information 3

Supplementary information 4

## Data Availability

All primary data is accessible at https://figshare.com/s/b694cc9ab2f1d31ae9c7 or 10.6084/m9.figshare.5657326.
