# Supplementary material for: Cadaverine, a metabolite of the microbiome, reduces breast cancer aggressiveness through trace amino acid receptors
Source: Sci Rep. 2019 Feb 4;9:1300. doi: 10.1038/s41598-018-37664-7 (PMC6361949; doi:10.1038/s41598-018-37664-7)
Supplement: Supplementary file 2 — Supplelemtary information 2 [file 41598_2018_37664_MOESM2_ESM.pdf]

Supplementary figure 2 for Kovács et al. "Cadaverine, a metabolite of the microbiome, reduces breast cancer aggressiveness through trace amino acid receptors"

Tünde Kovács[1], Edit Mikó[1,5], András Vida[1,5], Éva Sebő[6], Judit Toth[3], Tamás Csonka[4], Anita Boratkó[1], Gyula Ujlaki[1], Gréta Lente[1], Patrik Kovács[1], Dezső Tóth[3], Péter Árkosy[3], Borbála Kiss[2], Gábor Méhes[4], James J. Goedert[7], Péter Bai[1,5,8,*]

Departments of [1]Medical Chemistry, [2]Dermatology, [3]Oncology and [4]Pathology Faculty of Medicine, University of Debrecen, 4032, Hungary;

[5]MTA-DE Lendület Laboratory of Cellular Metabolism, Debrecen, 4032, Hungary;

[6]Kenézy Breast Center, Kenézy Gyula County Hospital, Debrecen, 4032, Hungary;

[7]National Cancer Institute, National Institutes of Health, Bethesda, 20982 MD, USA

[8]Research Center for Molecular Medicine, Faculty of Medicine, University of Debrecen, 4032, Hungary;

Running title: Cadaverine production modulates breast cancer

*Whom correspondence should be sent to:

Peter Bai, PhD, DSc University of Debrecen, Department of Medical Chemistry, 4032 Debrecen, Egyetem tér 1., Hungary, Tel. +36 52 412 345; Fax. +36 52 412 566, e-mail: baip@med.unideb.hu

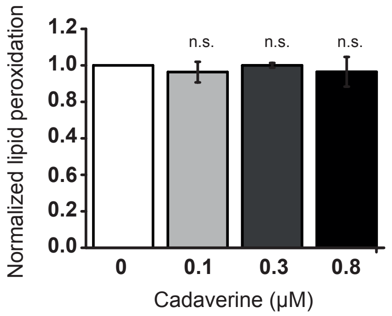
Normalized lipid peroxidation
1.2
1.0
0.8
0.4
0.6
0.2
0.0
n.s.
n.s.
n.s.
0
0.1
0.3
0.8
Cadaverine (μM)
