# Supplementary material for: Cadaverine, a metabolite of the microbiome, reduces breast cancer aggressiveness through trace amino acid receptors
Source: Sci Rep. 2019 Feb 4;9:1300. doi: 10.1038/s41598-018-37664-7 (PMC6361949; doi:10.1038/s41598-018-37664-7)
Supplement: Supplementary file 4 — Supplementary information 4 [file 41598_2018_37664_MOESM4_ESM.docx]

**Supplementary materials for Kovács et al. “Cadaverine, a metabolite of the microbiome, reduces breast cancer aggressiveness through trace amino acid receptors”**

Tünde Kovács^1^, Edit Mikó^1,5^, András Vida^1,5^, Éva Sebő^6^, Judit Toth^3^, Tamás Csonka^4^, Anita Boratkó^1^, Gyula Ujlaki^1^, Gréta Lente^1^, Patrik Kovács^1^, Dezső Tóth^3^, Péter Árkosy^3^, Borbála Kiss^2^, Gábor Méhes^4^, James J. Goedert^7^, Péter Bai^1,5,8,*^

Departments of ^1^Medical Chemistry, ^2^Dermatology, ^3^Oncology and ^4^Pathology Faculty of Medicine, University of Debrecen, 4032, Hungary;

^5^MTA-DE Lendület Laboratory of Cellular Metabolism, Debrecen, 4032, Hungary;

^6^Kenézy Breast Center, Kenézy Gyula County Hospital, Debrecen, 4032, Hungary;

^7^National Cancer Institute, National Institutes of Health, Bethesda, 20982 MD, USA

^8^Research Center for Molecular Medicine, Faculty of Medicine, University of Debrecen, 4032, Hungary;

Running title: Cadaverine production modulates breast cancer

*Whom correspondence should be sent to:

Peter Bai, PhD, DSc University of Debrecen, Department of Medical Chemistry, 4032 Debrecen, Egyetem tér 1., Hungary, Tel. +36 52 412 345; Fax. +36 52 412 566, e-mail: [baip@med.unideb.hu](mailto:baip@med.unideb.hu)

**Supplementary methods**

**Sulphorhodamine B assay**

Cells were seeded in a 96-well plates (4T1- 1500 cells/well; MDA-MB-231 and ZR-75-1- 3000 cells/well; SK-BR-3; MCF7 and human fibroblast- 5000 cells/well) and were let to attach overnight. Cells were treated with different concentration of cadaverine (Sigma Aldrich, C8561) for 48 hours. After 2 days cells were fixed by the addition of 50 % trichloroacetic acid (TCA, final concentration: 10 %) and the plate was incubated for 1 hour at 4 °C. The plate was then washed 5 times with water and was stained with 0.4 % (w/v) sulphorhodamine B solution in 1 % acetic acid. Unbound dye was removed by washing 5 times with 1 % acetic acid. Bound stain was solubilized with 10 mM Tris base and the absorbance was measured at 540 nm.

**Colony formation assay**

Cells were seeded in a 6-well plates (4T1- 750 cells/well; MDA-MB-231, SKBR-3, ZR-75-1 and MCF-7- 1000 cells/well) in complete medium and were treated with the indicated concentrations of cadaverine for 4 days. At the end of the treatment plates were washed in PBS. Colonies were fixed in 4 % PFA for 30 minutes, dried and stained with the solution of May-Grünwald-Giemsa for 30 minutes. Plates were washed with water and the colonies, containing at least 50 cells, were counted using Image J software.

**Detection of cell death**

For PI uptake cells were seeded in 6-well plate (MDA-MB-231, ZR-75-1 and MCF-7- 100000 cells/well; SKBR-3 and human fibroblast- 200000 cells/well). After 2 days of cadaverine treatment cells were stained with 100 µg/ml propidium iodide for 30 min at 37 °C. Supernatant was collected in FACS tubes, cells were washed with PBS and collected in the same FACS tubes (trypsin: PBS 1:1) then samples were analyzed by flow cytometry (FACS Calibur, BD Biosciences).

4T1 cells were seeded in 6-well plates (50000 cells/well) and treated with the indicated cadaverine concentrations for 2 days. Cells were harvested in FACS tubes, washed once with cold PBS and stained with 100 µg/ml PI solution and 5 µl FITC Annexin V (Component A) according to the instructions of the apoptosis kit. The number of apoptotic cells was measured with flow cytometry.

**Electric Cell-substrate Impedance Sensing (ECIS)**

4T1 cells were seeded on type 8W10E arrays (20000 cells/well) then treated with 0.1 µM cadaverine. ECIS (Electric cell-substrate impedance sensing) model Zθ, Applied BioPhysics Inc. (Troy, NY, USA) was used to monitor transcellular electric resistance of control and cadaverine treated cells for 20 hours before the treatment, and total impedance values were measured for additional 48 hours upon the indicated cadaverine treatment. Multifrequency measurements were taken at 62.5, 125, 250, 500, 1000, 2000, 4000, 8000, 16000, 32000, 64000 Hz. The reference well was set to a no-cell control with complete medium.

**SDS-PAGE and Western blotting**

Protein extracts were separated on 8% SDS polyacrylamide gels and transferred onto nitrocellulose membranes by electroblotting. Then membranes were blocked with 5 % BSA, and incubated with anti-Lysine decarboxylase primary antibody (1:100, Abcam), anti-TAAR1 polyclonal antibody (1:1000, Thermo Fisher Scientific), anti-TAAR8 polyclonal antibody (1:1000, Thermo Fisher Scientific) or anti-TAAR9 polyclonal antibody (1:1000, Biorbyt) for overnight at 4 °C. The membranes were washed with 1X TBS-TWEEN and incubated with IgG HRP conjugated secondary antibody (1:2000, Cell Signaling Technology). Bands were visualized by enhanced chemiluminescence reaction (SuperSignal West Pico Solutions, Thermo Fisher Scientific, 34578) using Chemidock Touch system. Densitometry was performed using the Image J software. Primary and secondary antibodies, used in this study, are listed in **Supplementary** **Table 2**.

**Scratch assay**

4T1 cells were plated in 6-well plates (150000 cells/well) and were grown overnight. The plates were manually scratched with sterile 200 µl pipette tip, followed by washing the cells with complete growth medium. Then cells were treated with 0.1 µM cadaverine in a 37°C thermostat and were monitored every hour for 36 hours using JuLi Br Live cell movie analyzer (NanoEnTek Inc., Seoul, Korea).

**Determination of lipid peroxidation (TBARS)**

Lipid peroxidation was measured by determining the production rate of thiobarbituric acid reactive substrate (TBARS). 4T1 cells were seeded in T75 flasks and allowed to adhere overnight. Cells were exposed to cadaverine for 48 hours, then collected by centrifugation. 8.1 % SDS, 20 % acetic acid, 0.8 % thiobarbituric acid (TBA) and distilled water was added to the pellet and was heated at 96°C for 1 hour in thermoblock. Samples were cooled on ice and centrifuged, the absorbance of the supernatant was measured at 540 nm.

**Transfections**

MDA-MB-231 cells were seeded on 24-well plate with or without coverslip, in 50000 cells/well density. Next day, cells were treated with vehicle or 0.3 μM cadaverine while transfected with human TAAR1 siRNA (Thermo Fisher Scientific, s223306) in 10 nM final concentration, human TAAR8 siRNA (Thermo Fisher Scientific, s223302) or human TAAR9 siRNA (Thermo Fisher Scientific, s38008) in 30 nM final concentration, or with control siRNA (Thermo Fisher Scientific, 4390843) in 30 nM final concentration. siRNAs were diluted in OptiMEM (Thermo Fisher Scientific, 31985062). 3 μl RNAiMAX transfection reagent (Thermo Fisher Scientific,13778) was used in each well according to the manufacturer’s instructions. Cells were incubated in 37°C with 5% CO_2_ for 48 hours. Then MMP9 and TexasRed-X Phalloidin immunocytochemistry were carried out on cells on coverslip, and Western blot analysis was carried out on cells without coverslip.

**mRNA isolation and quantitation**

Total RNA from cells were prepared using TRIzol reagent (Invitrogen, TR118). 2 µg RNA was reverse transcribed using High Capacity cDNA Reverse Transcription Kit (Applied Biosystems, Foster City, CA, USA, 4368813) according to the manufacturer’s instructions. qPCRBIO SyGreen Lo-ROX Supermix (PCR Biosystems Ltd, London, UK, PB20.11-05) was used for the qPCR reactions, the expression level of the genes was detected with Light-Cycler 480 Detection System (Roche Applied Science). Geometric mean of 36B4 and cyclophyllin or GAPDH was used for normalization. Primers are listed in **Supplementary** **Table 3**.

**Immunocytochemistry**

4T1 cells were grown on coverslips, and treated with the indicated concentration of cadaverine for 48 hours. To investigate the effect of TAARs, 5µM NF449 (Bio-Techne R&D Systems Kft, 627034-85-9) G-protein inhibitor was also added to cadaverine-treated cells. Cells were washed with PBS, fixed with 4 % PFA for 15 minutes and permeabilized using 1 % Triton X-100 for 5 minutes. After washing twice with PBS, cells were blocked with 1 % BSA for one hour at room temperature. For direct labelling of the actin cytoskeleton, fixed cells were incubated with TexasRed-X Phalloidin (1:150; Thermo Fisher Scientific) for an hour, followed by several washing steps with PBS. For visualizing MMP9 protein in 4T1 cells, MMP9 primary antibody (1:1000, Abcam) was applied overnight on cells in a humid chamber at 4 ^o^C. Subsequently, primary antibody was visualized by an goat anti-rabbit secondary antibody (1:500, Thermo Fisher Scientific). Cell nuclei were visualized with TO-PRO-3 iodide (1:1000, Thermo Fisher Scientific), or DAPI (1:10, Thermo Fisher Scientific). Coverslips were rinsed and mounted in Mowiol/Dabco solution. Confocal images were acquired with Leica SP8 confocal microscope and LAS AF v3.1.3 software. Intensity was calculated using Image J software, Cell Profiler 2.0 or Advanced Cell Classifier 3.0. Materials used in immunocytochemistry assays are summarized in **Supplementary** **Table 2**.

**Validating of *E. coli* LdcC antibody**

DH5α *Escherichia coli* were seeded in liquid LB medium. Cells were incubated at 37°C overnight with gentle shaking. *E. coli* cells were then collected with centrifugation and proteins were isolated using RIPA buffer (50 mM Tris, 150 mM NaCl, 0.1 % SDS, 1 % TritonX 100, 0.5 % sodium deoxycholate, 1 mM EDTA, 1 mM Na_3_VO_4_, 1 mM NaF, 1 mM PMSF, protease inhibitor cocktail). Cell wall and membrane were disrupted using ultrasound sonicator (Branson Ultrasonic Sonifier S-250A, Thermo Fisher Scientific) 3 times for 30 seconds at 50 % amplitude. Samples were analyzed by SDS-PAGE followed by Western blotting (**Fig. S3**).

**Aldefluor assay**

Aldehyde dehydrogenase (ALDH) activity was determined on cadaverine treated 4T1 and MDA-MB-231 cells using Aldefluor Stem Cell kit (StemCell Technologies, Vancouver, Canada, #01700) Cells were seeded on 6 well plates (4T1- 50000 cells/well) or on 24 well plates (MDA-MB-231- 50000 cells/well) and treated with different concentration of cadaverine for 2 days. Cells were then collected and prepared according to the manufacturer’s instructions. We used SKBR-3 cell line for positive control. Changes in the level of ALDH was measured using flow cytometry and the results were analyzed with flowing software 2.5.1.

**Supplementary Tables**

**Supplementary Table 1. Primers for the determination of the abundance of CadA and LdcC using RT-qPCR**

| **Organism and gene name** | **Forward primer (5’-3’)** | **Reverse primer (5’-3’)** |
| --- | --- | --- |
| Escherichia coli LdcC | cggcccttataacctgctgtttc | ccttgtgccagatcctgaatacg |
| Escherichia coli CadA | gtctgtgcggcgttatttttgac | cacccagcgcatattcaaagaag |
| Enterobacter cloacae LdcC | atatgatctgaacctgcgggtga | aggttctccagctcaacggtttc |
| Hafnia alvei LdcC | ggtgaactgggttctctgcttga | agcgggtgctgagtacataccaa |

**Supplementary Table 2. List of antibodies used for Western blot /Immunocytochemistry**

| **Antibody** | **Concentration** | **Vendor** |
| --- | --- | --- |
| anti MMP9 antibody | 1:1000 | Abcam (ab38898) |
| Anti-lysine decarboxylase, constitutive antibody | 1:100 | Abcam (ab193351) |
| anti-rabbit IgG, HRP-linked antibody | 1:2000 | Cell Signaling Technology (#7074) |
| Goat anti-Rabbit IgG (H+L) Cross-Adsorbed Secondary Antibody (Alexa Fluor 488) | 1:500 | Thermo Fisher Scientific (A11008) |
| Texas Red™-X Phalloidin | 1:250 | Thermo Fisher Scientific (T7471) |
| TO-PRO®-3 | 1:1000 | Thermo Fisher Scientific (T3605) |
| DAPI | 1:10 | Thermo Fisher Scientific (R37606) |
| TAAR1 polyclonal antibody | 1:1000 | Thermo Fisher Scientific (PA5-23141) |
| TAAR8 polyclonal antibody | 1:1000 | Thermo Fisher Scientific (OSR00118W) |
| TAAR9 polyclonal antibody | 1:1000 | Biorbyt LLC (orb165536) |

**Supplementary Table 3. Primers used in the RT-qPCR reactions**

| **Gene Symbol** | **Murine forward primer (5’-3’)** | **Murine reverse primer (5’-3’)** |
| --- | --- | --- |
| MMP2 | tgggggagattctcactttg | catcactgggaccagtgtct |
| MMP3 | tgggactctaccactcagccaag | tgcacattggtgatgtctcaggt |
| MMP9 | cattcgcgtggataaggagt | acctggttcacctcatggtc |
| Krt14 | gaagaggccaacactgaactgga | aggctctgctccgtctcaaactt |
| Spp1 | gattggcagtgatttgcttttgc | ttctgcttctgagatgggtcagg |
| FgfBp1 | caaggtccaagaagctgtctcca | agctccaagattccccacagaac |
| Notch1 | ccttcacctgtctgtgtccacct | tcacagtggtactgcgtgttggt |
| Tgfb3 | ggcgtctcaagaagcaaaaggat | ccttaggttcgtggacccatttc |
| Erbb3 | tacttgcctctgggctctctcct | cacctggacttgactcggtgact |
| Er1 | gaccatgacccttcacaccaaag | ctcggggtagttgaacacagtgg |
| IgfBp4 | caagatgaagatcgtggggacac | cagtttggaatggggatgatgaa |
| 36B4 | AGATTCGGGATATGCTGTTGG | AAAGCCTGGAAGAAGGAGGTC |
| CyclophillinA | TGGAGAGCACCAAGACAGACA | TGCCGGAGTCGACAATGAT |
| GAPDH | CAAGGTCATCCATGACAACTTTG | GGCCATCCACAGTCTTCTGG |

**Supplementary Table 4.**

| **Significant changes in gene expression of 4T1 cells and tumor samples** | | |
| --- | --- | --- |
|  |  |  |
| **4T1 cells** |  |  |
| **Gene** | **Groups** | **P-value** |
| MMP3 | control and 0.3 µM CAD | **0.048** |
| MMP3 | control and 0.8 µM CAD | **0.033** |
| MMP9 | control and 0.1 µM CAD | **7 x 10^-3^** |
| MMP9 | control and 0.3 µM CAD | **9 x 10^-3^** |
| Tgfb3 | control and 0.1 µM CAD | **9 x 10^-3^** |
| Tgfb3 | control and 0.3 µM CAD | **9.47 x 10^-5^** |
| Tgfb3 | control and 0.8 µM CAD | **1.96 x 10^-5^** |
| FgFbp1 | control and 0.3 µM CAD | **1 x 10^-3^** |
| FgFbp1 | control and 0.8 µM CAD | **1.21 x 10^-4^** |
| Erbb3 | control and 0.8 µM CAD | **0.037** |
| Er1 | control and 0.3 µM CAD | **0.035** |
| Notch1 | control and 0.3 µM CAD | **4 x 10^-3^** |
| Notch1 | control and 0.8 µM CAD | **1 x 10^-3^** |
| IgFbp4 | control and 0.1 µM CAD | **9.45 x 10^-5^** |
| IgFbp4 | control and 0.3 µM CAD | **2.27 x 10^-4^** |
| IgFbp4 | control and 0.8 µM CAD | **6.83 x 10^-6^** |
| Spp1 | control and 0.3 µM CAD | **0.024** |
| Spp1 | control and 0.8 µM CAD | **0.032** |
| **Metastases** |  |  |
| **Gene** | **Groups** | **P-value** |
| MMP3 | VEH and CAD-treated | **0.027** |
| CDH1 | VEH and CAD-treated | **0.041** |
| DSC2 | VEH and CAD-treated | **0.046** |

**Figure legends to Supplementary Figures**

**Supplementary Figure 1. Cadaverine reverts cancer hallmarks in breast cancer cell lines**

**(A)** In control and cadaverine-treated MDA-MB-231 (n=1 in triplicates) breast cancer cells morphology of the actin cytoskeleton was assessed after Texas Red-X Phallodin and DAPI staining. Ratio (%) of epithelial and mesenchymal cells was shown on bar charts. Significance was calculated using Chi-square test in Microsoft Excel.

**(B)** MDA-MB-231 cells (n=2, triplicates) were treated with vehicle and cadaverine for 48 hours, then cells were stained for MMP9 and nucleus (DAPI) and sections were analyzed by confocal microscopy using a Leica SP8 confocal system. MMP9 content was calculated from the total cellular fluorescence measured by Cell Profiler 2.0 software. Kruskal-Wallis statistical test was carried out using Graphpad 7.0 software.

**(C)** MDA-MB-231 cells (n=2, duplicates) were treated with vehicle and cadaverine for 48 hours, then cells were stained with Aldefluor kit and was analyzed by FACS. Two sample t-test was carried out on results.

(D) In control and cadaverine-treated SKBR-3 (n=1 in triplicates) breast cancer cells morphology of the actin cytoskeleton was assessed after Texas Red-X Phallodin and DAPI staining. Ratio (%) of epithelial and mesenchymal cells was shown on bar charts. Significance was calculated using Chi-square test in Microsoft Excel.

**(E)** SKBR-3 cells (n=2, triplicates) were treated with vehicle and cadaverine for 48 hours, then cells were stained for MMP9 and nucleus (DAPI) and sections were analyzed by confocal microscopy using a Leica SP8 confocal system. MMP9 content was calculated from the total cellular fluorescence measured by Cell Profiler 2.0 software. Kruskal-Wallis statistical test was carried out using Graphpad 7.0 software.

* and *** indicate statistically significant difference between control and cadaverine treated groups at p<0.05 and p<0.01, respectively.

**Supplementary Figure 2. Cadaverine does not influence lipid peroxidation**

Lipid peroxidation was measured by determining the production rate of thiobarbituric acid reactive substrate (TBARS). 4T1 cells were treated with different concentration of cadaverine for 48 hours. 8.1 % SDS, 20 % acetic acid, 0.8 % thiobarbituric acid (TBA) and distilled water was added to the pellet and was heated at 96°C. Absorbance of the supernatant was measured at 540 nm. (n=3 triplicates) n.s. – not significant

**Supplementary Figure 3. Validation of the E. coli LdcC antibody**

DH5α E. coli cells were grown in LB. After incubation overnight cells were collected and lysed in RIPA buffer. Samples were separated using SDS-PAGE (20, 30 and 50 µg protein extract were loaded) followed by Western blotting. Membranes were blocked with 5 % BSA and LdcC antibody (Abcam) was applied overnight on the membranes, for visualization HRP-linked secondary antibody (Cell Signaling Technology) was used. Blots were routinely cut as on the sample image.
